# Increasing game prices may alter farmers’ behaviours towards leopards (*Panthera pardus*) and other carnivores in South Africa

**DOI:** 10.7717/peerj.3369

**Published:** 2017-05-30

**Authors:** Tara J. Pirie, Rebecca L. Thomas, Mark D.E. Fellowes

**Affiliations:** 1 People and Wildlife Research Group, School of Biological Sciences, University of Reading, Reading, Berkshire, United Kingdom; 2 Ingwe Leopard Research, Lydenburg, Mpumalanga, South Africa; 3 School of Biological Sciences, Royal Holloway University of London, Egham, Surrey, United Kingdom

**Keywords:** Human-wildlife conflict, Leopards, Carnivore conservation, Conservation economics, *Panthera pardus*, Retaliation killings, Livestock

## Abstract

Human-carnivore conflict occurs globally, particularly in regions where large carnivores predate livestock. Retaliatory killings do occur, and although predation of livestock by carnivores happens, losses from other factors such as disease or injury can be misattributed because of landowner perceptions. Game farming for both trophy hunting and eco-tourism is becoming increasingly common in South Africa, and there has been a rapid increase in the cost of game animals (in some species as much as five-fold) between 2010 and 2015. This could result in an increase in conflict between commercial game farmers and carnivores. We conducted two questionnaire surveys of farmers in 2010 and 2015 to investigate this. We asked if there had been changes in farming practices, perceived predator activity, perceived amount of livestock and commercial game losses, and actions taken towards carnivores in a South African farming community. We found no significant change in farming types in the area or losses of livestock between the years. However, there was a significant increase in perceived commercial game losses reported, even though protection of game had increased. Actions taken towards carnivores by livestock/game farmers were also significantly more negative in 2015 compared to farmers growing crops, but there was no such difference in 2010. We suggest that these changes could be a result of the increase in game prices over that period, leading to greater financial losses when an animal is predated, which in turn could increase the likelihood of retaliatory killings of carnivores.

## Introduction

As human populations increase, human-wildlife conflict grows, particularly when human livelihoods are negatively affected by wildlife (Thirgood, Woodroffe & Rabinowitz, 2005; Dickman, 2010). The increased encroachment of people into wilderness areas is inevitable (Asibey, 1974; Sillero-Zubiri & Switzer, 2001) and limited resources often force wildlife into areas of pasture or arable land where conflict can occur (Sillero-Zubiri & Switzer, 2001; Thirgood, Woodroffe & Rabinowitz, 2005; Athreya et al., 2007; Dickman, 2010). Conflicts arise for many reasons and with many taxa, including larger mammalian carnivores (e.g., tiger *Panthera tigris*, Himalayan black bear *Ursus thibetanus*, snow leopard *Uncia uncia*, leopard *Panthera pardus*; Sangay & Vernes, 2008; wolves *Canis lupus,* lynx *Lynx lynx,* brown bear *Ursus arctos*; Kaczensky, 1999) and herbivores (e.g., elephant *Loxodonta africana,* bushpig *Potamochoerus porcus*; Naughton-Treves, 1998). Human-wildlife conflict will undoubtedly continue to be a key factor in the decline of wildlife populations, particularly for carnivores (Woodroffe, 2000; Dickman, Glen & Letnic, 2009; Hoffman & O’Riain, 2012), unless issues can be highlighted and addressed.

Predators can often be wrongly accused of livestock predation, and losses may actually occur from theft (Rust et al., 2016), injury, disease, poor nutrition, or venomous snake bites (Polisar et al., 2003). Nevertheless, large mammalian carnivores can pose a real threat to livestock (Yom-Tov, Ashkenazi & Viner, 1995; Meriggi & Lovari, 1996; Wagner, & Conover, 1999; Odden et al., 2002; Sunquist & Sunquist, 2002; Bagchi & Mishra, 2006; Garrote et al., 2013), particularly where natural prey abundance is low (Polisar et al., 2003; Kolowski & Holekamp, 2006; Boast et al., 2016). This can lead to the removal of problem animals either legally or illegally, due to financial losses incurred (Thorn et al., 2013), but removal may also occur because of the perceived threat that carnivores pose (Sangay & Vernes, 2008; Balme, Slowtow & Hunter, 2009). Removal methods such as trapping, relocation (Linnell et al., 1997; Athreya, 2006; Weilenmann et al., 2010), and lethal control (Treves & Naughton-Treves, 2005) have been used to alleviate problems, however ecological consequences such as meso-predator release and trophic cascades caused by the elimination of apex predators have been documented across numerous systems (Crooks & Soulé, 1999; Schmitz, Hambäck & Beckerman, 2000; Letnic & Koch, 2010). Methods such as the use of guard dogs or donkeys *Equus africanus asinus* (Ogada et al., 2003; Gehring et al., 2010), retaining horns on cows, mixing heifers with older and more experienced cows, synchronised calving, using calving camps and electric fencing (Reinhardt et al., 2012; Lindsey et al., 2013), or even using groups of adults rather than boys as herders (Svengren & Björklund, 2010), have been used in an attempt to prevent livestock depredation rather than removing carnivores from an area.

Compensation schemes, which reimburse farmers for damage caused by large carnivores in the hope of reducing negativity towards predators and therefore retaliatory killings when losses occur, have been attempted globally across European countries (Boitani, Ciucci & Raganella-Pelliccioni, 2011; Rigg et al., 2011), India, the USA (Agarwala et al., 2010), and South Africa (Anthony, Scott & Antypas, 2010). While some areas have not noted any change in negative attitudes towards predators or increases in predator numbers (Agarwala et al., 2010; Boitani, Ciucci & Raganella-Pelliccioni, 2011), the state of Wisconsin, USA, initiated a scheme in 1982 and recorded a significant increase in grey wolf *Canis lupus* numbers over 30 years (Treves et al., 2009), although the increase was relatively low, suggesting that illegal killings were still occurring (Chapron & Treves, 2016). Conservation performance payments have also been trialled in Sweden, whereby successful carnivore reproduction earned reindeer *Rangifer tarandus* herders a reward. The payment was calculated based on the number of carnivore offspring and the future damage these animals were predicted to cause (Zabel & Holm-Müller, 2008). This scheme has shown potential, and was associated with a local increase in wolverine *Gulo gulo* numbers over 10 years (Zabel, Bostedt & Engel, 2014).

Improving habitats for prey species and “farming” wildlife has also been recommended as an effective way to reduce predation of livestock (Winterbach et al., 2015; Boast et al., 2016), while generating income through tourism and promoting the conservation of carnivores (Lindsey et al., 2013). Game farming has increased rapidly in South Africa, from an estimated 5,000 farms in 2003 (Carruthers, 2008) to almost 9,000 in 2016 (Taylor et al., 2016) and these are often located in the same areas as livestock farms (Thorn et al., 2012; Swanepoel et al., 2014; Pitman et al., 2016). Generally the game farms are used for tourism (e.g., photography, hunting) and/or breeding economically valuable species including sable *Hippotragus niger*, roan antelope *Hippotragus equinus* and disease free African buffalo *Syncerus caffer* (Taylor et al., 2016).

Although there are pockets of wild lion *Panthera leo*, cheetah *Acinonyx jubatus* and wild dog *Lycon pictus*, in South Africa, these predators mainly reside within protective game fences (Hayward & Kerley, 2009; Stuart & Stuart, 2015); however leopard (Balme, Slowtow & Hunter, 2009; Swanepoel, Somers & Dalerum, 2015), brown hyena *Hyaena brunnea* (Mills, 1982), and meso-predators such as caracal and jackal species are commonly found outside reserves (Thorn et al., 2011; Kingdon, 2011), and therefore are more likely to come into conflict with farmers. Annual livestock deaths due to black-backed jackal and caracal are thought to be considerable; one estimate suggests losses of up to R1.4 billion (US$98 million, Bergman et al., 2013). Other studies have also shown leopard and brown hyena are often held responsible for livestock losses in South Africa (Kissui, 2008; St John, Edwards-Jones & Jones, 2010; St John et al., 2011; Thorn et al., 2012; Yirga et al., 2012). Damage causing animal (DCA) permits may be requested to remove problem animals which are approved by the provincial authorities (Pitman et al., 2016), however in many South African provinces DCA permits are not required to remove black-backed jackal or caracal. Furthermore, illegal retaliatory killings of IUCN red data listed species such as leopard or brown hyena have been reported (Balme & Hunter, 2004; St John, Edwards-Jones & Jones, 2010; St John et al., 2011; Thorn et al., 2012).

Conflict between livestock farmers and carnivores has been well documented (Kaczensky, 1999; Polisar et al., 2003; Sangay & Vernes, 2008; St John, Edwards-Jones & Jones, 2010; St John et al., 2011; Thorn et al., 2012) although the potential conflict with game farmers, specifically game breeders, has not been investigated to the same degree. Boast (2014) reported that game farmers were more tolerant of predators than livestock farmers, but it has been suggested that increased game prices may reduce differences in attitudes (Pitman et al., 2016).

Retaliatory killings are believed to have a greater detrimental impact on local populations than regulated removal (Swanepoel et al., 2014), therefore understanding the occurrence of retaliatory killings and the causal reasons for conflict is important. The game farming industry has expanded rapidly in South Africa (Taylor et al., 2016), but relatively little is understood about potential conflict with predators. We conducted surveys of farmers in 2010 and 2015 to ascertain if farming practices, perceived predator activity and perceived losses of livestock and/or game were associated with negative actions taken towards carnivores, and use the data to ask if the occurrence of negative actions had changed over the time period of the study, which was conducted on the border between the Limpopo and Mpumalanga provinces, South Africa.

**Figure 1:**
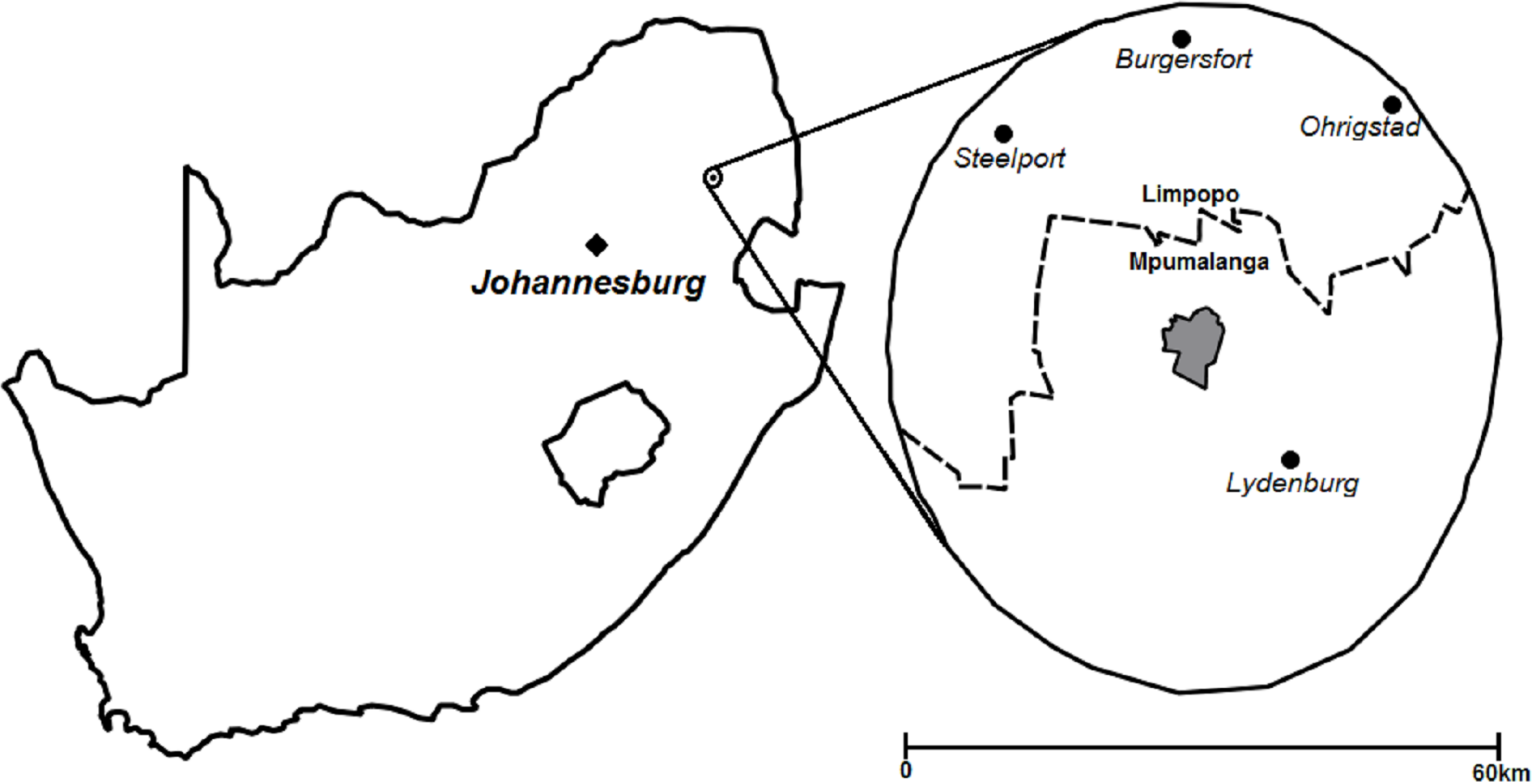
Location of area surveyed within 30 km of Thaba Tholo Wilderness Reserve (grey), South Africa. Dashed line represents the provincial boundary between Limpopo and Mpumalanga, Quantum GIS 2.16.2, 19 April 2017.

## Materials and Method

Identical surveys were conducted in 2010 and 2015 which engaged with farmers who managed land over 20 ha in area and were located within a 30 km buffer zone of the Thaba Tholo Wilderness Reserve where our research site was based. Thaba Tholo holds good populations of large carnivores, including leopard (Pirie, Thomas & Fellowes, 2016a; Pirie, Thomas & Fellowes, 2016b). This area included the towns and regions of Steelport, Burgersfort, Lydenburg and Ohrigstad in Limpopo and Mpumalanga provinces, South Africa (Fig. 1). This selection process incorporated farmers mainly from an Afrikaans speaking background who are the major landowners in the region.

The aim of the 2015 survey was to replicate the 2010 surveys with the same farmers using the contact information gathered during the 2010 process. However some farmers were no longer present in the area or could not be contacted (due to family illness or death, or change in contact details). To compensate for this, farmers new to the area were questioned along with other farmers who were not part of the original survey. The interviews were conducted by a female Afrikaans speaking researcher either by telephone or in person and took an average of 15 minutes to complete. Before the survey was conducted the respondent was informed the survey was being undertaken by the researcher on behalf of Ingwe Leopard Research and the University of Reading and asked if they would be willing to take part in the survey about carnivores on their property. If they consented they were asked if they would prefer to take part over the telephone or face to face; 30 chose the former with five opting for the latter in 2015; unfortunately this information was not available for the 2010 surveys.

The original 2010 survey was based on a standard survey used by the Endangered Wildlife Trust (1973), which was in turn replicated in 2015 and was approved by the University of Reading ethics committee (School of Biological Sciences #SBS 13–14 19). The survey contained five sections of mainly closed-ended questions, which asked: (A) general information about the farm size and location; (B) type of farm (crops, livestock, wildlife), type of water source and terrain; (C) infrastructure and management of livestock and game (monitored, guarded, placed in overnight camps or in camps permanently); (D) perceived predator activity recorded as sightings or spoor. The section also included an open question asking for perceived losses of livestock/game per year which were recorded as species lost and the perceived reason for the loss. The final section (E) asked about action taken towards carnivore species, including leopard, brown hyena, caracal, jackal sp., serval, civet, genet sp., and feral dog. While civets and genets are unlikely to attack young ungulates they will attack domestic fowl and small mammals. Options for actions taken against each predator were as follows; no opinion, shoot, poison, trap, tolerate (no action taken although would prefer no carnivore presence), friendly (no action taken and the farmer welcomes the presence of carnivores) and other. For the analysis the overall behaviours towards carnivores were categorised based on how the respondent reacted to each carnivore species. Positive meant that the respondent gave all “friendly” or “tolerant” responses to each carnivore hence no action would be taken towards any carnivore; both was used when there was a mixture of “friendly” or “tolerant” responses with shoot/trap/poison; negative was used when no friendly or tolerant responses were given but either shoot/trap/poison was given for all or most carnivore species. At the end of the 2015 questionnaire, respondents were asked an open ended question which required them to state if their behaviour towards carnivores had changed over the last five years, regardless of whether they had participated in the previous survey or not, and, why this was so.

### Statistical analyses

In the second survey a proportion of farmers had also answered the first questionnaire. We therefore analysed the data in two ways. First, we considered unmatched data, where for individuals who had answered both questionnaires only the second (2015) data set was used, ensuring that the analysis was performed on two separate groups of farmers in each survey year. Second, we performed a matched analysis using just the data from those who had answered both questionnaires. Where appropriate, multiple tests were adjusted using Holm’s sequential Bonferroni corrections (Holm, 1979).

#### Unmatched surveys—all farmers

Mann–Whitney–Wilcoxon tests were used to investigate whether a change had occurred in farming practice and animal management, losses experienced and behaviour towards carnivores in the area over the five year period and Fisher’s exact tests were used to investigate any differences between years for a given question. Two farm owners had two separate properties in 2015 which were treated as separate farms for all the questions except for that concerned with action taken. Crop farmers were defined as growing crops only, but may have had wildlife naturally on the property. Livestock farmers were defined as farming domestic livestock although may have grown crops, and/or had wildlife or commercial game. Commercial game farmers were defined as farming/breeding game to sell rather than using for photography purposes, although they may have livestock, crops and/or natural game as well. Species reared included kudu *Tragelaphus strepsiceros*, nyala *Tragelaphus angasii*, bushbuck *Tragelaphus scriptus,* Burchell’s zebra *Equus quagga burchelli*, both wildebeest *Connochaetes* spp., blesbok *Damaliscus pygargus phillipsi*, impala *Aepyceros melampus* and colour variants of these species.

#### Matched surveys—stock farmers only

A Wilcoxon signed-rank test was used to compare whether farming practice had changed between the same farmers, if there was any change in levels of losses and whether responder actions towards carnivores had changed between surveys.

## Results

In 2010 we had a response rate of 90% which yielded a total of 63 completed surveys and a response rate of 47% in 2015 with 35 completed surveys. Response rates were lower in 2015 due to the difficulty in contacting original participants. Most respondents were farmers, with two managers of citrus farms and four spouses responding in 2015. Twenty of the surveys conducted in 2015 were with the same individual farmers as 2010, with the remaining fifteen undertaken with new respondents whose details were given by other farmers or from the farm road signs outside the properties.

**Table 1:** Percentages of responses from closed-ended question categories. Farm type is defined as Crop only (only crops, no animal stock), Livestock (has domestic stock, although may farm crops or have natural wildlife), Game (farm/breed game for selling, although also may have livestock, crops or natural wildlife) and Wildlife (has wildlife naturally on the property but does not commercially farm). In addition farm size, provision of protection for livestock and game and behaviour towards all focus carnivores are reported for all farms (2010 *N* = 43, 2015 *N* = 35).

Question	Response category	2010%	2015%
Farm type	Crop only	19 (*N* = 8)	26 (*N* = 9)
	Livestock	56 (*N* = 24)	63 (*N* = 22)
	Game	7 (*N* = 3)	22 (*N* = 9)
	Wildlife	18 (*N* = 8)	0 (*N* = 0)
Size of farm	<300 ha	35 (*N* = 15)	29 (*N* = 10)
	300–1,000 ha	28 (*N* = 12)	31 (*N* = 11)
	>1,000 ha	26 (*N* = 11)	26 (*N* = 9)
	No comment	11 (*N* = 5)	14 (*N* = 5)
Protection	Yes	92 (*N* = 22)	65 (*N* = 17)
	No	8 (*N* = 2)	35 (*N* = 9)
Behaviour	Positive	49 (*N* = 21)	45 (*N* = 15)
	Both	44 (*N* = 19)	30 (*N* = 10)
	Negative	5 (*N* = 2)	19 (*N* = 6)
	No comment	2 (*N* = 1)	6 (*N* = 2)

### Unmatched surveys

**Figure 2:**
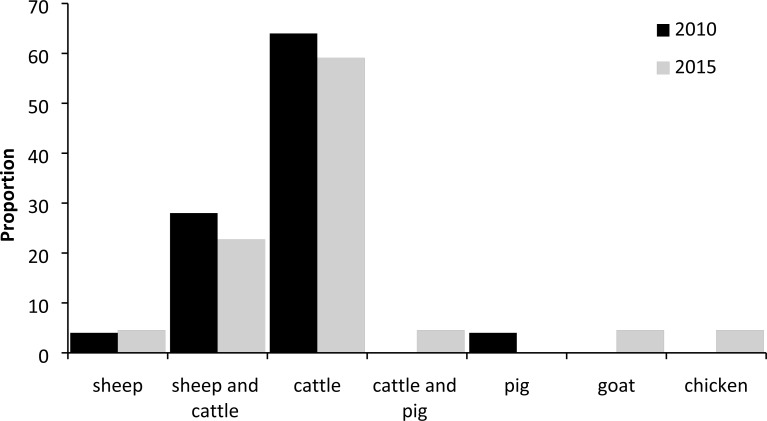
Percentage of different types of livestock reared in surveyed farms, 2010 and 2015.

In comparing the respondents who were questioned independently in 2010 (*N* = 43) and 2015 (*N* = 35) there was very little change between most responses. Farm type was classified as crop (2010 *N* = 8, 2015 *N* = 9), livestock (2010 *N* = 24, 2015 *N* = 22) and commercial game farmers (2010 *N* = 3, 2015 *N* = 9). In 2010 there were eight farms with natural wildlife only. Of the 78 farms in total, 84% had wild game on the property. Although there was a significant difference between all property types initially (*W* = 514, 2010 *N* = 43, 2015 *N* = 35, *p* = 0.04), this became non-significant when only commercial farming was considered (i.e., properties with only wild game were removed, Table 1). Cattle formed the majority of farmed livestock (Fig. 2) and farm size was variable (<300 ha: 32%; between 300 ha and 1,000 ha: 28%; over 1,000 ha: 26%). There was a significant decrease in any form of animal protection by stock farmers, either by providing overnight camps, guarding by people or dogs, or monitoring young animals (*W* = 218, 2010 *N* = 22, 2015 *N* = 16, *p* = 0.014; Table 1); however, although numbers were small, there was a significant increase in protection of commercial game in the form of fenced camps in 2015 (Fisher’s exact test 2010 *N* = 0, 2015 *N* = 7, *p* = 0.045, Table 2).

**Table 2:** Percentages of responses for unmatched surveys from livestock (2010 *N* = 24, 2015 *N* = 22) and commercial game farmers (2010 *N* = 3, 2015 *N* = 9) regarding animal protection, perceived losses, and action taken towards different carnivore species over both years (2010 *N* = 25, 2015 *N* = 24).

Question	Response category	2010%	2015%
Protection	Livestock yes	88 (*N* = 21)	59 (*N* = 13)
	Commercial game yes	0 (*N* = 0)	78 (*N* = 7)
Losses	Livestock	79 (*N* = 19)	81 (*N* = 18)
	Commercial game	33 (*N* = 1)	100 (N=9)
Removal of species	Leopard	16 (*N* = 4)	29 (*N* = 7)
	Hyena	20 (*N* = 5)	25 (*N* = 6)
	Jackal sp.	40 (*N* = 10)	54 (*N* = 13)
	Caracal	4 (*N* = 1)	25 (*N* = 6)
	Feral dog	40 (*N* = 10)	21 (*N* = 5)
Evidence of species	Leopard	83 (*N* = 20)	91 (*N* = 20)
	Hyena	67 (*N* = 16)	73 (*N* = 16)
	Jackal sp.	75 (*N* = 18)	95 (*N* = 21)
	Caracal	54 (*N* = 13)	64 (*N* = 14)

Although there was no significant difference in perceived predator activity between both years (Table 2), a significant increase was found in reported animal losses in 2015 of commercial and wild animals (*W* = 531.5, 2010 *N* = 43, 2015 *N* = 35, *p* = 0.02). Further investigation found livestock loss was not significantly different but losses of wildlife, wild or commercial, to predators were significantly different between years (Fisher’s exact test 2010 *N* = 2, 2015 *N* = 10, adjusted *p* = 0.017), with 8% of farms in 2010 (*N* = 38) reporting losses compared to 36% in 2015 (*N* = 28). Commercially farmed game losses alone had increased significantly in 2015 (Fisher’s exact test 2010 *N* = 1, 2015 *N* = 9, *p* = 0.045, Table 2).

Both leopard and caracal were thought to be the cause of more losses in 2015 by both livestock and commercial game farmers (Table 3), however only responses for the believed caracal-caused losses were found to be statistically significantly higher (*W* = 603.5, 2010 *N* = 43, 2015 *N* = 35, adjusted *p* = 0.045). There was no significant difference in action taken towards the carnivores between both years for all respondents, with 46% of the total farms surveyed (*N* = 78) responding positively towards carnivores compared with 47% who responded negatively towards one or more predator (Table 1). However, livestock and commercial game farmers responded significantly more negatively than agricultural farmers in 2015 (*W* = 56.5, total stock farmers *N* = 26, non-stock farmers *N* = 9, *p* = 0.033). Conversely there were no significant differences between stock farmers or agricultural farmers in 2010.

**Table 3:** Percentage of responses for unmatched surveys on the perceived causes of stock losses in both years broken down into livestock and commercial game farmers. (Two livestock farmers had more than one farm in 2015 therefore *N* = 20.) Total percentages incorporated total farmers for the year and perceptions regardless of type of animal stock farmed.

Perceived cause of loss	Livestock % 2010 (*N* = 24)	Livestock % 2015 (*N* = 20)	Commercial game % 2010 (*N* = 3)	Commercial game % 2015 (*N* = 9)	Total % 2010 (*N* = 25)	Total % 2015 (*N* = 24)
Leopard	17 (*N* = 4)	30 (*N* = 6)	0	44 (*N* = 4)	16	67
Brown hyena	21 (*N* = 5)	25 (*N* = 5)	0	11 (*N* = 1)	20	25
Caracal	0	10 (*N* = 2)	0	22 (*N* = 2)	0	16
Jackal sp.	42 (*N* = 10)	25 (*N* = 5)	0	44 (*N* = 4)	40	42
Other carnivore	4 (*N* = 1)	0	0	0	16	0
Feral dogs	0	0	0	11 (*N* = 1)	0	4
Poachers	8 (*N* = 2)	5 (*N* = 1)	33 (*N* = 1)	22 (*N* = 2)	12	13
Disease	4 (*N* = 1)	0	0	0	16	0
Unsure	13 (*N* = 3)	25 (*N* = 5)	33 (*N* = 1)	22 (*N* = 2)	16	4
Remove one or more carnivore species	58 (*N* = 14)	59 (*N* = 10)	33 (*N* = 1)	67 (*N* = 6)	60	66

### Matched analyses—stock farmers only

There were 20 repeated surveys of which two farmed only crops in both years; these were omitted from further analyses. There was no difference in animal management (monitored, guarded, placed in overnight camps or in camps permanently), however losses were found to have significantly increased in 2015 (*W* = 11, *N* = 18, *p* = 0.037). Actions taken towards carnivores were not found to significantly differ between the surveys.

### Changes in behaviours

When farmers were asked if their behaviour towards carnivores had changed in the 2015 surveys (*N* = 33), 23 stated their behaviour remained unchanged, with fifteen continuing to be positive. Of the ten whose behaviour had reportedly changed, two farmers had replied they had become “more negative”, one “due to the substantial loss he has sustained (40 nyala at R 30,000 (US$2,058) per animal) over the last four years”. Eight stated that they had become more positive, two of whom said they had become “better informed” and “better educated to the fact they have a place”. One farmer had become more positive because “they do not farm with cattle anymore”. Most had replied there was no change. One farmer “accepted it was part of farming,” however some would “allow a certain amount (of losses) but then I need to remove the problem, but there is no-one to help with this,” and others responded that if they find predators are creating a big problem they will shoot them but they did not want to shoot them. Another responded that if the predator attacks too much of the stock they would “put methods in place to fix it, like shoot them”. Two farmers had commented that they thought there was an “increase in the carnivore populations and activity due to towns increasing in size and therefore less land is available for the animals”. Two farmers commented on their perceived increase in the use of snares and one farmer disclosed that he knew of ten leopards which had been illegally killed in the area over the last ten years because of livestock losses.

## Discussion

While we acknowledge that survey numbers are low and therefore present a limitation to the study, there are patterns which have emerged highlighting potential issues in the future. There was a significant increase in reported total animal losses between our surveys of 2010 and 2015, which could be a result of the decrease in animal protection and management seen across the survey period. However perceived predation of livestock from the independent surveys did not change significantly during the five year period but results suggest that game losses did increase. Even though the commercial farming of game species was not found to have significantly increased in the area, results show that the protection of game, in the form of fenced camps, also increased in 2015. While action taken against carnivores did not change between years, there was a significant difference between livestock/commercial game farmers and agricultural farmers in 2015, with the former responding less favourably towards carnivores. There was however no difference in 2010 between the stock and non-stock farmers, suggesting there may have been a change which was not highlighted due to relatively small numbers; indeed, although not significant, a higher percentage of commercial game farmers in 2015 responded they would remove one or more predator species compared to livestock farmers.

Attitudes have often been used to investigate human-carnivore conflict in order to predict potential negative behaviour towards predators (Agarwala et al., 2010; Boitani, Ciucci & Raganella-Pelliccioni, 2011), but concerns have been raised about this approach (Wallace et al., 2005; Gross, 2016; St John, Edwards-Jones & Jones, 2010). The main argument is that attitudes are considered to be part of behavioural belief (see Ajzen & Madden, 1986; Ajzen, 1991; St John, Edwards-Jones & Jones, 2010), which is only part of the process motivating actual behaviour. Other factors such as social pressure, culture and beliefs are also believed to contribute to and influence the end behaviour (Wallace et al., 2005; Gross, 2016) and often surveys do not consider other factors which may influence actions (St John, Edwards-Jones & Jones, 2010) which may be different to predictions based on attitudes (St John, Edwards-Jones & Jones, 2010; Thorn et al., 2012). It has however been demonstrated that actual behaviour is more likely to correspond with intended behaviour (Albarracin et al., 2001). Therefore in this study behaviour categories are based on actions taken against each carnivore; we asked responders about actions they had taken towards carnivores, rather than their attitudes, to allow us to gauge their views of predators. In addition, although perceptions of loss and actual losses are not always aligned (Boast et al., 2016), people’s perceptions can be enough to trigger retaliation (Mishra, 1997; Madden, 2004; Sangay & Vernes, 2008; Balme, Slowtow & Hunter, 2009; Dickman, 2010), therefore we were interested in what farmers perceived to be the reality about predator activity and animal losses in this study. At the same time, we must acknowledge that by asking questions concerning potentially illegal behaviours, we may be biasing results, as individuals are less likely to respond honestly (or at all) to such queries (Nuno & St John, 2015). Therefore our results may be conservative where issues of human-wildlife conflict are considered.

As a result we did not evaluate losses caused by predator activity, but instead considered actions taken against predators by farmers (Dickman, 2010). A decrease in management of livestock in the area was reported over the study period, but there was no significant change in losses perceived to be caused by large predators. However, commercial game losses were significantly more likely to occur in 2015 despite the suggestion that protective camps were more frequently used in 2015. Although fences are designed to separate species farmed from wildlife (Woodroffe, Hedges & Durant, 2014), they are not always flawless (Hoare, 1992; Cozzi et al., 2013). African wild dogs have been documented using fences to increase hunting success (Davies-Mostert, Mills & Macdonald, 2013). Ogada et al. (2003) found that livestock kept in kraals overnight were less likely to be attacked by predators such as the lion, leopard and spotted hyena *Crocuta crocuta*, but this may be a consequence of the presence of watch dogs and high levels of human activity, rather than the presence of the barrier to predator movement.

The increase in reported individual game losses may have simply been due to a rise in predation (albeit farmers did not perceive a change in predator activity), or due to reduced access to wild game. However it may be that the commercial game farmers in 2015 acknowledged losses more due to the initial cost of the stock and potentially the inflation in the monetary value of game, causing a greater perceived financial loss occurring from the death of each individual animal compared to previous years (Table 4). Live game sales in South Africa were thought to generate an annual turnover revenue of R4.328 billion in 2014 (US$ 296 million, Taylor et al., 2016), a four-fold increase since 2012 (Pitman et al., 2016). Species such as sable *Hippotragus niger* had an increase in purchase cost of 470% between 2009 and 2014 and disease free African buffalo increased in value over 540%; one bull alone was sold for R40 million (US$2.7 million, Anonymous, 2015). Prices of animals such as nyala and impala have also increased dramatically, with colour variants receiving particularly high prices (Table 4) which are mainly sold for trophy hunting (Taylor et al., 2016), rather than photographic tourism. Conversely, there has been little change in the cost of live cattle (Table 4; Anonymous, 2016), or beef and mutton prices (Janovsky, 2013) over the last five years.

**Table 4:** Indicative auction prices of game and cattle in South Africa for 2011, 2013 and 2015. US$ value calculated at an exchange rate of 14.31 Rand to the US$.

Animal	Colour	Cost 2011	Ref.	Cost 2013	Ref.	Cost 2015	Ref.
Nyala	normal	R6,809 (US$476)	Anonymous (2013)	R10,706 (US$748)	Anonymous (2013)	R21,205 (US$1,482)	Erasmus (2016)
Impala	normal	R1,106 (US$77)	Anonymous (2013)			R2,568 (US$179)	Erasmus (2016)
Impala	black	R230,000 (US$16,077)	Erasmus (2016)			R275,400 (US$19,251)	Erasmus (2016)
Impala	white	R330,000 (US$23,068)	Erasmus (2016)			R2 million (US$139,803)	Erasmus (2016)
Cattle		R18.36/kg^a^ (US$1.28)	Anonymous (2016)	R18.28/kg (US$1.28)	Anonymous (2016)	R19.68/kg (US$1.38)	Anonymous (2016)

**Notes.**

a Data for January 2012.

Pitman et al. (2016) found a significant positive correlation in the number of DCA permit requests to remove carnivores and numbers of commercial game farmers in the Limpopo province over nine years, however no DCA permits were issued by the provincial authorities in Mpumalanga during the study period (J Muller, pers. comm., 2016). Nevertheless, retaliatory killings were divulged by some respondents during the survey, and although some farmers acknowledged they would kill leopard and other carnivores, true actions may not have been admitted due to the directness of the question. However, this has given some insight into the potential for removal of carnivores which would have gone unnoticed if DCA permit allocations were used alone to assess the situation.

While we acknowledge that the questionnaire response rate is low, particularly from commercial game owners, there was a documented increase in the management and protection of game, which could be a reflection of their increasing economic value (a trend also seen in Limpopo Province in the only similar study that we are aware of; Pitman et al., 2016). One game farmer acknowledged that increased financial losses meant that he had become more negative towards carnivores, a major motivator of retaliatory killings in other studies (Bagchi & Mishra, 2006; Kissui, 2008). It is worth noting that many who said they would remove problem animals acknowledged that they did not want to do so, suggesting that they would be open to other strategies, which may indicate that retaliatory killings could be reduced with the right approach to support farmers. Supporting this, other farmers noted that negative action had reduced due to them viewing carnivores in a more positive light, understanding that carnivores “had a place”.

## Conclusion

Farms changed little over the five year interval between surveys, and there was no change in perceived losses of domestic livestock thought to be caused by carnivores, or in numbers of commercial game farmers. Perceived increases in losses of commercial game animals may be associated with the rapid rise in the financial value of game animals relative to that of livestock, which may be reflected by the significant increase in game protection in the form of fenced camps. It is therefore plausible that the significant difference in anti-predator actions reported in 2015 between stock (livestock and commercial game) and non-stock farmers may be due to increased game prices. Stock farmers understandably show more negative behaviour towards carnivores than non-stock farmers, as they experience direct financial loss when stock is taken (Thorn et al., 2013).

Our study suggests that retaliation killings do occur in the study area, despite animals such as leopard and brown hyena being protected and that no DCA permits were issued over the previous ten years (J Muller, pers. comm., 2016). This illustrates the importance of quantifying illegal carnivore removals for insights into local levels of human-wildlife conflict. Retaliatory killings may increase in the future if game value continues to rise and the financial loss per animal is increased, which should be acknowledged as a developing threat to South Africa’s carnivores. For example, at one study site in South Africa’s Limpopo Province, leopard numbers declined by 66% between 2012 and 2016. This was in large part attributed to illegal killing, including retaliation for perceived livestock predation (Williams et al., 2017). Therefore we advise that further consideration should be given to understanding the implications of this growing industry on carnivores across South Africa.

##  Supplemental Information

10.7717/peerj.3369/supp-1 Data S1 Pirie et al data Responses to questionnaires received from farmers, 2010 and 2015. Each farm is uniquely coded. Responses were either yes(y)/no(n)/not completed (nc) or quantitative.

## Additional Information and Declarations

### Data Availability

The following information was supplied regarding data availability: The raw data have been supplied as Data S1.

## References

[ref-1] Agarwala M, Kumar S, Treves A, Naughton-Treves L (2010). Paying for wolves in Solapur, India and Wisconsin, USA: comparing compensation rules and practice to understand the goals and politics of wolf conservation. Biological Conservation.

[ref-2] Ajzen I (1991). The theory of planned behavior. Organizational Behavior and Human Decision Processes.

[ref-3] Ajzen I, Madden TJ (1986). Prediction of goal-directed behavior: attitudes, intentions, and perceived behavioral control. Journal of Experimental Social Psychology.

[ref-4] Albarracin D, Johnson BT, Fishbein M, Muellerleile PA (2001). Theories of reasoned action and planned behavior as models of condom use: a meta-analysis. Psychological Bulletin.

[ref-5] (2013). Average auction prices 2011 to 2013. http://oscar.caxtonmagsapps.co.za/img/uploads/file/Farmer's%20Weekly/2014/Average%20auction%20prices_2011%20to%202013pdf.

[ref-6] (2015). Wildlife auctions, big bucks at stake. http://www.financialmail.co.za/features/2015/08/20/wildlife-auctions-big-bucks-at-stake.

[ref-7] (2016). Market indicators. http://www.safeedlot.co.za/index.asp?Content=151.

[ref-8] Anthony BP, Scott P, Antypas A (2010). Sitting on the fence? Policies and practices in managing human-wildlife conflict in Limpopo Province, South Africa. Conservation and Society.

[ref-9] Asibey OE (1974). Wildlife as a source of protein in Africa south of the Sahara. Biological Conservation.

[ref-10] Athreya V (2006). Is relocation a viable management option for unwanted animals? The case of the leopard in India. Conservation and Society.

[ref-11] Athreya VR, Thakur SS, Chaudhuri S, Belsare AV (2007). Leopards in human-dominated areas: a spillover from sustained translocations into nearby forests. Journal of the Bombay Natural History Society.

[ref-12] Bagchi S, Mishra C (2006). Living with large carnivores: predation on livestock by the snow leopard (*Uncia uncia*). Journal of Zoology.

[ref-13] Balme G, Hunter L (2004). Mortality in a protected leopard population, Phinda Private Game Reserve, South Africa: a population in decline. Ecological Journal.

[ref-14] Balme GA, Slowtow R, Hunter LTB (2009). Impact of conservation interventions on the dynamics and persistence of a persecuted leopard (*Panthera pardus*) population. Biological Conservation.

[ref-15] Bergman DL, De Waal H, Avenant NL, Bodenchuk M, Marlow MC, Nolte DL, Armstrong JB, Gallagher GR (2013). The need to address black-backed jackal and caracal predation in South Africa.

[ref-16] Boast L (2014). Exploring the causes of and mitigation options for human-predator conflict on game ranches in Botswana: how is coexistence possible?. Doctoral dissertation.

[ref-17] Boast L, Houser A, Horgan J, Reeves H, Phale P, Klein R (2016). Prey preferences of free ranging cheetahs on farmland: scat analysis versus farmers’ perceptions. African Journal of Ecology.

[ref-18] Boitani L, Ciucci P, Raganella-Pelliccioni E (2011). Ex-post compensation payments for wolf predation on livestock in Italy: a tool for conservation?. Wildlife Research.

[ref-19] Carruthers J (2008). Wilding the farm or farming the wild? The evolution of scientific game ranching in South Africa from the 1960s to the present. Transactions of the Royal Society of South Africa.

[ref-20] Chapron G, Treves A (2016). Blood does not buy goodwill: allowing culling increases poaching of a large carnivore. Proceedings of the Royal Society B Biological Sciences.

[ref-21] Cozzi G, Broekhuis F, McNutt JW, Schmid B (2013). Comparison of the effects of artificial and natural barriers on large African carnivores: implications for interspecific relationships and connectivity. Journal of Animal Ecology.

[ref-22] Crooks KR, Soulé ME (1999). Mesopredator release and avifaunal extinctions in a fragmented system. Nature.

[ref-23] Davies-Mostert HT, Mills MGL, Macdonald DW (2013). Hard boundaries influence African wild dogs’ diet and prey selection. Journal of Applied Ecology.

[ref-24] Dickman AJ (2010). Complexities of conflict: the importance of considering social factors for effectively resolving human—wildlife conflict. Animal Conservation.

[ref-25] Dickman CR, Glen AS, Letnic M, Hayward MW, Somers MJ (2009). Reintroducing the dingo: can Australia’s conservation wastelands be restored?. Reintroduction of top-order predators.

[ref-26] Endangered Wildlife Trust (1973). https://endangeredwildlifetrust.wordpress.com/about-theewt/.

[ref-27] Erasmus (2016). Gemiddelde wildveilingpryse: 2013–2015. http://www.gamefarmnet.co.za/veiling.htm.

[ref-28] Garrote G, López G, Gil-Sánchez JM, Rojas E, Ruiz M, Bueno JF, De Lillo S, Rodriguez-Siles J, Martín JM, Pérez J (2013). Human–felid conflict as a further handicap to the conservation of the critically endangered Iberian lynx. European Journal of Wildlife Research.

[ref-29] Gehring TM, VerCautreren KC, Provost ML, Cellar AC (2010). Utility of livestock-protection dogs for deterring wildlife from cattle farms. Wildlife Research.

[ref-30] Gross J (2016). Do attitudes predict behaviours? The jaguar and its allies. https://thejaguarandallies.com/2016/04/21/do-attitudes-predict-behaviors/.

[ref-31] Hayward MW, Kerley GIH (2009). Fencing for conservation: restriction of evolutionary potential or a riposte to threatening processes?. Biological Conservation.

[ref-32] Hoare RE (1992). Present and future use of fencing in the management of larger African mammals. Environmental Conservation.

[ref-33] Hoffman TS, O’Riain MJ (2012). Monkey management: using spatial ecology to understand the extent and severity of human baboon conflict in the Cape Peninsula, South Africa. Ecology and Society.

[ref-34] Holm S (1979). A simple sequentially rejective multiple test procedure. Scandinavian Journal of Statistics.

[ref-35] Janovsky E (2013). Looking at livestock prices. http://www.grainsa.co.za/looking-at-livestock-prices-for-2014.

[ref-36] Kaczensky P (1999). Large carnivore depredation on livestock in Europe. Ursus.

[ref-37] Kingdon J (2011). The Kingdon field guide to African mammals.

[ref-38] Kissui B (2008). Livestock predation by lions, leopards, spotted hyenas, and their vulnerability to retaliatory killing in the Maasai steppe, Tanzania. Animal Conservation.

[ref-39] Kolowski JM, Holekamp KE (2006). Spatial, temporal, and physical characteristics of livestock depredations by large carnivores along a Kenyan reserve border. Biological Conservation.

[ref-40] Letnic M, Koch F (2010). Are dingoes a trophic regulator in arid Australia? A comparison of mammal communities on either side of the dingo fence. Australian Ecology.

[ref-41] Lindsey PA, Havemann CP, Lines R, Palazy L, Price AE, Retief TA, Rhebergen T, Van der Waal C (2013). Determinants of persistence and tolerance of carnivores on Namibian ranches: implications for conservation on Southern African private lands. PLOS ONE.

[ref-42] Linnell JDC, Aanes R, Swenson JE, Odden J, Smith ME (1997). Translocation of carnivores as a method for managing problem animals: a review. Biodiversity & Conservation.

[ref-43] Madden F (2004). Creating coexistence between humans and wildlife: global perspectives on local efforts to address human-wildlife conflict. Human Dimensions of Wildlife.

[ref-44] Meriggi A, Lovari S (1996). A review of wolf predation in southern Europe: does the wolf prefer wild prey to livestock?. Journal of Applied Ecology.

[ref-45] Mills MGL (1982). Hyaena brunnea. Mammalian Species.

[ref-46] Mishra C (1997). Livestock depredation by large carnivores in the Indian trans-Himalaya: conflict perceptions and conservation prospects. Environmental Conservation.

[ref-47] Naughton-Treves L (1998). Predicting patterns of crop damage by wildlife around Kibale National Park, Uganda. Conservation Biology.

[ref-48] Nuno A, St John FAV (2015). How to ask sensitive questions in conservation: a review of specialized questioning techniques. Biological Conservation.

[ref-49] Odden J, Linnell JD, Moa PF, Herfindal I, Kvam T, Andersen R (2002). Lynx depredation on domestic sheep in Norway. Journal of Wildlife Management.

[ref-50] Ogada MO, Woodroffe R, Oguge NO, Frank LG (2003). Limiting depredation by African carnivores: the role of livestock husbandry. Conservation Biology.

[ref-51] Pirie TJ, Thomas RL, Fellowes MDE (2016a). Erythristic morphs of the leopard *Panthera pardus* in South Africa. Bothalia: African Biodiversity and Conservation.

[ref-52] Pirie TJ, Thomas RL, Fellowes MDE (2016b). Relative efficacy of camera trap and spoor count data for recording mammalian predators in African savannah. Wildlife Biology.

[ref-53] Pitman RT, Fattebert J, Williams ST, Williams KS, Hill RA, Hunter LT, Slotow R, Balme GA (2016). The conservation costs of game ranching. Conservation Letters.

[ref-54] Polisar J, Maxit I, Scognamillo D, Farrell L, Sunquist ME, Eisenberg JF (2003). Jaguars, pumas, their prey base, and cattle ranching: ecological interpretations of a management problem. Biological Conservation.

[ref-55] Reinhardt I, Rauer G, Kluth G, Kaczensky P, Knauer F, Wotschikowsky U (2012). Livestock protection methods applicable for Germany—a country newly recolonized by wolves. Hystrix.

[ref-56] Rigg R, Finďo S, Wechselberger M, Gorman ML, Sillero-Zubiri C, Macdonald DW (2011). Mitigating carnivore—livestock conflict in Europe: lessons from Slovakia. Oryx.

[ref-57] Rust NA, Tzanopoulos J, Humle T, MacMillan DC (2016). Why has human-carnivore conflict not been resolved in Namibia?. Society & Natural Resources.

[ref-58] Sangay T, Vernes K (2008). Human–wildlife conflict in the Kingdom of Bhutan: patterns of livestock predation by large mammalian carnivores. Biological Conservation.

[ref-59] Schmitz OJ, Hambäck PA, Beckerman AP (2000). Trophic cascades in terrestrial systems: a review of the effects of carnivore removals on plants. American Naturalist.

[ref-60] Sillero-Zubiri C, Switzer D (2001). Crop raiding primates: searching for alternative, humane ways to resolve conflict with farmers in Africa.

[ref-61] St John FAV, Edwards-Jones G, Jones JP (2010). Conservation and human behaviour: lessons from social psychology. Wildlife Research.

[ref-62] St John FAV, Keane AM, Edwards-Jones G, Jones L, Yarnell RW, Jones JP (2011). Identifying indicators of illegal behaviour: carnivore killing in human-managed landscapes. Proceedings of the Royal Society of London B: Biological Sciences.

[ref-63] Stuart C, Stuart M (2015). Field guide to mammals of southern Africa.

[ref-64] Sunquist M, Sunquist F (2002). Wild cats of the world.

[ref-65] Svengren H, Björklund M (2010). An assessment of the density of a large carnivore using a non-invasive method adapted for pilot studies. South African Journal of Wildlife Research.

[ref-66] Swanepoel LH, Somers MJ, Dalerum F (2015). Density of leopards *Panthera pardus* on protected and non-protected land in the Waterberg Biosphere, South Africa. Wildlife Biology.

[ref-67] Swanepoel LH, Somers MJ, Van Hoven W, Schiess-Meier M, Owen C, Snyman A, Martins Q, Senekal C, Camacho G, Boshoff W, Dalerum F (2014). Survival rates and causes of mortality of leopards *Panthera pardus* in Southern Africa. Oryx.

[ref-68] Taylor A, Lindsey P, Davies-Mostert H, Goodman P (2016). An assessment of the economic, social and conservation value of the wildlife ranching industry and its potential to support the green economy in South Africa. Green Economy Research Report.

[ref-69] Thirgood S, Woodroffe R, Rabinowitz A, Woodroffe R, Thirgood S, Rabinowitz A (2005). The impact of human-wildlife conflict on human lives and livelihoods. People and wildlife: conflict or coexistence?.

[ref-70] Thorn M, Green M, Dalerum F, Bateman PW, Scott DM (2012). What drives human-carnivore conflict in the North West province of South Africa?. Biological Conservation.

[ref-71] Thorn M, Green M, Keith M, Marnewick K, Bateman PW, Cameron EZ, Scott DM (2011). Large-scale distribution patterns of carnivores in northern South Africa: implications for conservation and monitoring. Oryx.

[ref-72] Thorn M, Green M, Scott DM, Marnewick K (2013). Characterisitics and determinants of human-carnivore conflict in South African farmland. Biodiversity and Conservation.

[ref-73] Treves A, Jurewicz RL, Naughton-Treves L, Wilcove DS (2009). The price of tolerance: wolf damage payments after recovery. Biodiversity and Conservation.

[ref-74] Treves A, Naughton-Treves L, Woodroffe R, Thirgood S, Rabinowitz A (2005). Evaluating lethal control in the management of human-wildlife conflict. People and wildlife: conflict or coexistence?.

[ref-75] Wagner KK, Conover MR (1999). Effect of preventive coyote hunting on sheep losses to coyote predation. Journal of Wildlife Management.

[ref-76] Wallace DS, Paulson RM, Lord CG, Bond Jr CF (2005). Which behaviors do attitudes predict? Meta-analyzing the effects of social pressure and perceived difficulty. Review of General Psychology.

[ref-77] Weilenmann M, Gusset M, Mills DR, Gabanapelo T, Schiess-Meier M (2010). Is translocation of stock-raiding leopards into a protected area with resident conspecifics an effective management tool?. Wildlife Research.

[ref-78] Williams ST, Williams KS, Lewis BP, Hill RA (2017). Population dynamics and threats to an apex predator outside protected areas: implications for carnivore management. Royal Society Open Science.

[ref-79] Winterbach HE, Winterbach CW, Boast LK, Klein R, Somers MJ (2015). Relative availability of natural prey versus livestock predicts landscape suitability for cheetahs *Acinonyx jubatus* in Botswana. PeerJ.

[ref-80] Woodroffe R (2000). Predators and people: using human densities to interpret declines of large carnivores. Animal Conservation.

[ref-81] Woodroffe R, Hedges S, Durant SM (2014). To fence or not to fence. Science.

[ref-82] Yirga G, De Iongh HH, Leirs H, Gebrehiwot K, Berhe G, Asmelash T, Gebrehiwot H, Bauer H (2012). The ecology of large carnivores in the highlands of northern Ethiopia. African Journal of Ecology.

[ref-83] Yom-Tov Y, Ashkenazi S, Viner O (1995). Cattle predation by the golden jackal *Canis aureus* in the Golan Heights, Israel. Biological Conservation.

[ref-84] Zabel A, Bostedt G, Engel S (2014). Performance payments for groups: the case of carnivore conservation in Northern Sweden. Environmental and Resource Economics.

[ref-85] Zabel A, Holm-Müller K (2008). Conservation performance payments for carnivore conservation in Sweden. Conservation Biology.

